# Mr. Ernest Richard Evans

**Published:** 1911-05-20

**Authors:** 


Mr. Ernest Richard Evans, M.R.C.S., has died at Hertford, aged sixty-six. He studied at St. Bartholomew's Hospital, took his M.R.C.S. in 1867, and his L.R.C.P. and L.S.A. in 1870. He had been house surgeon and house physician at St. Bartholomew's, resident medical officer at the Evelina Hospital for Sick Children, and later was medical visitor at Hadham Palace Asylum. Mr. Evans was consulting medical officer to the Hertford General Infirmary.

